# Impact of CD68, CD4, TNF-α, and COX-2 expression on disease-specific survival in Brazilian patients with OSCC

**DOI:** 10.1590/1807-3107bor-2025.vol39.121

**Published:** 2025-11-17

**Authors:** Sibele Nascimento de AQUINO, Lucas Lacerda de SOUZA, Hélen Kaline Farias BEZERRA, Daniel Gomes de ALVARENGA, Paulo Rogério Ferreti BONAN, Helder Domiciano Dantas MARTINS, Alan Roger SANTOS-SILVA, Márcio Ajudarte LOPES, Pablo Agustin VARGAS

**Affiliations:** (a) Universidade Federal de Juiz de Fora – UFJF, Applied Health Sciences Post-Graduate Program, Governador Valadares, MG, Brazil.; (b) Universidade Estadual de Campinas – Unicamp, Piracicaba Dental School, Department of Oral Diagnosis, Piracicaba, SP, Brazil.; (c) Universidade Federal de Juiz de Fora – UFJF, Departament of Medicine, Governador Valadares, MG, Brazil.; (d) Universidade Federal da Paraíba – UFPb, Post-Graduate Program in Dentistry, João Pessoa, Brazil.

**Keywords:** Squamous Cell Carcinoma of Head and Neck, Cyclooxygenase 2, Pathology, Mouth Neoplasms, Prognosis

## Abstract

Oral squamous cell carcinoma (OSCC) is the most common malignancy of the head and neck. Studies on the inflammatory pathways that have evolved during the development of the disease remain controversial. We assessed the expression of inflammatory markers, such as COX (cyclooxygenase)-2, CD68, CD4, and tumor necrosis factor (TNF)-α, based on prognostic variables and disease-specific survival in patients with OSCC. Immunohistochemical analysis of COX-2, TNF-α, CD4, and CD68 was conducted in 72 patients treated surgically. Neural invasion was evaluated based on S100 expression. Disease-specific survival was assessed using Cox regression analysis. Most participants were male, with a mean age of 61 years. A total of 77.5% of patients presented with clinical stages III–IV, and 70% underwent surgery combined with radiotherapy or chemotherapy. The expression of CD68, CD4, and TNF-α was not associated with clinical variables or tumor differentiation. COX-2 expression correlated with tumor size (p = 0.01), whereas high TNF-α expression was noted in moderately/poorly differentiated OSCC. The absence of nodal involvement (hazard ratio [HR]: 0.47, confidence interval [CI]: 0.25–0.87, p = 0.001) was linked to lower death risk, whereas surgery without adjuvant radiotherapy or chemotherapy was associated with a higher risk of death (HR: 2.09, 95%CI: 1.02–4.27, p = 0.043). Multivariate analysis revealed that high COX-2 expression predicted a shorter disease-specific survival. Altogether, high TNF-α expression is prevalent in moderately/poorly differentiated OSCC, and elevated COX-2 expression correlates with larger tumor size and poorer survival in OSCC.

## Introduction

Oral squamous cell carcinoma (OSCC) is the most common type of malignant head and neck cancer. Tobacco smoking and alcohol consumption are recognized as its major causal factors. It is associated with a high rate of morbidity and mortality worldwide. Despite several advancements in therapeutic approaches, it remains a leading cause of death among men in several regions, with a 5-year survival rate of approximately 50%.^1^

Investigation of the molecular markers of OSCC provides new perspectives for the development of targeted therapeutics. Several markers are linked to cell survival and proliferation, angiogenesis, and immunosuppression within the tumor microenvironment (TME) of OSCC, including those associated with tumor-associated inflammatory cells. Inflammatory cells are vital components of the TME and influence the genesis, survival, proliferation, and metastasis potential of OSCC.^2^

CD68 is a macrophage marker, particularly M2-polarized tumor-associated macrophages (TAMs), which are associated with immunosuppression, extracellular matrix remodeling, and poor prognosis in OSCC. Their presence and function are shaped by tumor necrosis factor (TNF)-α signaling and cyclooxygenase (COX)-2 activity, reinforcing a tumor-promoting cycle.^3^ CD4+ T cells orchestrate adaptive immune responses and can play both antitumor (Th1) and pro-tumor (Th2/Treg) roles depending on the surrounding cytokine environment. Their function is modulated by TNF-α levels, which can either drive inflammation or contribute to immune evasion.^4,5^

TNF-α functions as a central inflammatory cytokine, linking chronic inflammation to cancer progression. It influences macrophage polarization (CD68) and enhances COX-2 expression, creating a feedback loop that sustains tumor-promoting inflammation.^6^ COX-2, a key inflammatory enzyme, promotes tumor growth, immune suppression, and angiogenesis. It influences the recruitment and activation of TAMs (marked by CD68) and contributes to an inflammatory microenvironment favoring tumor progression.^7,8^

Considering these findings and the controversies observed in previous studies, we evaluated the association of the CD68, CD4, TNF-α, and COX-2 with prognostic factors, including perineural invasion (PNI), and disease-specific survival in OSCC in a Brazilian sample.

## Methods

This retrospective study included 72 patients diagnosed with primary OSCC, who were surgically treated at a Cancer Center. Formalin-fixed paraffin-embedded (FFPE) samples were obtained from the Pathological Laboratory. This study was reviewed and approved by the Institutional Ethics Committee (#1.821.102).

Independent predictor variables included patient sex, age at diagnosis, primary tumor site, smoking and drinking status, regional lymph node status, clinical stage, metastasis, treatment modality, date of diagnosis, date of last consultation, and date and cause of death. The AJCC 8th edition was used for TNM classification.^9^ Patients with an incomplete clinical history (including time and cause of death) or insufficient tissue were excluded. In addition, patients diagnosed in the oropharyngeal region were excluded.

The tumors were microscopically reevaluated and classified as well-differentiated, moderately differentiated, or poorly differentiated.^10^ Immunohistochemistry was performed in 3-μm-thick sections from formalin-fixed, paraffin-embedded tissue blocks. Primary antibodies against CD68 (Santa Cruz; E-11 clone, sc-17832, lot # IO123, dilution 1:100), TNF-α (Santa Cruz, TN3-19.12 clone, sc12744, lot #B2823, dilution 1:100), CD4 (Novusbio; RPA-T4 clone, lot #AB061008A-3, dilution 1:100), and COX-2 (Santa Cruz, 29 clone, sc-1999, lot #E2223, dilution 1:100) were used. Multifocal neural invasion was evaluated by measuring S100 expression (Dako; polyclonal, lot #36387, dilution 1:10000). The tissue sections were deparaffinized and hydrated. Antigen retrieval was performed using the ethylenediaminetetraacetic acid/Tris solution (pH 9.0) or citric acid solution (pH 6.0) for 15 min in an electric pressure cooker. The endogenous peroxidase activity was blocked with 6% H_2_O_2_ (20 vol.) for 15 min. The tissue sections were incubated with the primary antibody for 2 h at room temperature. The detection system used was the Envision–Dual Link System-HRP (Dako; Carpinteria, USA), and staining was performed with diaminobenzidine (DAB; Dako) for 5 min. Counterstaining was performed using Carazzi’s hematoxylin. Tonsil tissue sections were used as positive controls.

Immunohistochemical quantification was performed using QuPath-0.5.0-x64 (University of Edinburgh, Scotland, UK).^11^ In summary, five images of each marker IHC slide were imaged at 20x magnification using a Leica DFC345 FX. The images were imported to QuPath-0.5.0-x64 (University of Edinburgh, Scotland, UK) as “Heme/DAB brightfield.” The images were annotated with rectangular annotations to avoid the background with brushes. The magnification and resolution of the photomicrographs were kept constant. RGB pixel depth stain vectors were recalibrated using the “Estimate Stain Vectors” tool before algorithmic counting, employing the default “auto” detection. Next, positive cell detection was used to select the sum of the optical densities. The general parameters were maintained, and the “Score Compartment” was set to be “Cytoplasm: DAB OD mean” or “Cell: DAB OD mean,” according to the marker. After analysis, the images with respective pixel overlays were reviewed to assess the adequacy of the thresholds and were adjusted if positive cells were not counted.

The expression of CD68, CD4, and TNF-α was analyzed in the stroma. Tumor and stromal expression were evaluated for COX-2. The mean expression of the marker was calculated, following which dichotomization was performed and the tumors were classified as Grade I and Grade II using the K-means to determine cutoff points for the categories.^12^ The extent of PNI was classified as unifocal or multifocal, depending on whether single or multiple PNI foci were observed.^13^ Almost all patients with PNI present with multifocal neural involvement. Considering the more robust data, multifocal neural involvement was selected as the predictor variable for this analysis.

**Figure 1:**
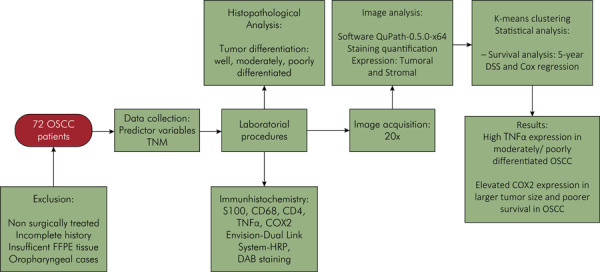
Flowchart detailing the methodological data of the study and the main results.

The data were summarized using crosstabs, and the associations between variables were assessed using the chi-square or Fisher’s exact test and the Mann–Whitney test. Survival analysis considered 5-year disease-specific survival (DSS). DSS was established based on the time between the date of diagnosis and the date of death due to OSCC. The Kaplan–Meier method was used to construct survival curves, which were compared using a log-rank test. A multivariate Cox regression model was created using the variable that achieved a p-value ≤ 0.20. Data were processed using the JAMOVI software (version 1.6.23), and the level of statistical significance was set at 5% (p < 0.05). Figure 1 presents a flowchart detailing the methodological data of the study and the main results.

## Results

Most participants were male (75%) and were diagnosed at over 50 years (80.6%) (mean age: 61.1, range: 30–93 years). A large proportion of participants were smokers (74.2%) and alcohol consumers (56.7%). Patients were more commonly diagnosed at clinical stages III and IV (77.5%). Regarding the treatment protocol, 69.8% of the patients were treated with surgery, radiotherapy, or chemotherapy. The tongue (50%) was the most common tumor location, followed by the floor of the mouth (23.6%). The tumor involved multiple sites, such as the tongue, floor of the mouth, and gingiva in eight cases (11.1%). PNI was observed in 36 cases (52,17%). In addition, unifocal occurrence was observed in 7 cases (19.4%) and multifocal occurrence in 29 cases (80.6%) (Table 1).

**Table 1:** Clinical characteristics of the study sample (n = 72).

Classification	n (%)
Age (years)	
≤ 50	14 (19.4)
≥ 51	58 (80.6)
Sex	
Male	54 (75.0)
Female	58 (19.4)
Smoking status	
Not smoker	16 (26.8)
Smoker	46 (74.2)
Drinking status	
Never-drinker	26 (43.3)
Drinker	34 (56.7)
Histological grading (WHO)	
Well-differentiated	37 (51.4)
Moderately/poorly differentiated	35 (48.6)
Tumor (T)	
1–2	26 (37.7)
3–4	43 (62.3)
Regional lymph nodes (N)	
No	32 (46.4)
Yes	37 (53.6)
Clinical stage	
I-II	16 (22.5)
III-IV	55 (77.5)
Radiotherapy (RT)	
No	18 (29.0)
Yes	44 (71.0)
Chemotherapy (CT)	
No	29 (49.2)
Yes	30 (50.8)
No	44 (69.8)
Yes	19 (30.2)
Surgery + RT or CT	
Surgery alone	
No	44 (61.1)
Yes	28 (38.9)
Tumor site	
Tongue	36 (50.0)
Floor of the mouth	17 (23.6)
Palate	6 (8.3)
Buccal mucous	2 (2.8)
Retromolar	3 (4.2)
Multiple site	8 (11.1)
Outcome	
Dead	48 (66.7)
Alive	24 (33.3)
Perineural invasion (PNI)	
Yes	36 (52.2)
No	32 (46.4)
Perineural invasion (PNI)	
Unifocal	7 (19.4)
Multifocal	29 (80.6)

**Figure 2:**
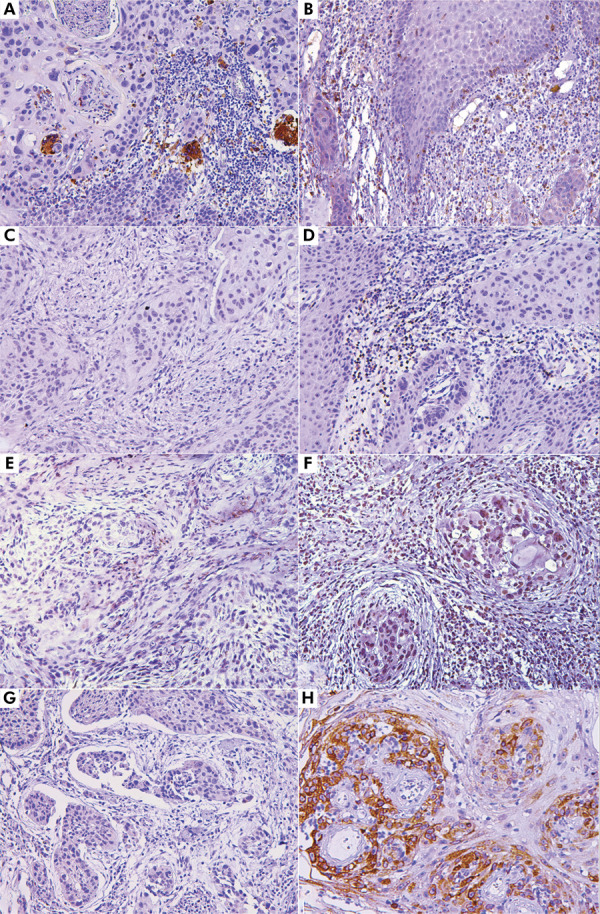
Photomicrographs of the immunohistochemical analysis, demonstrating variable expression of the markers analyzed. (A, B) Variable expression of CD68 (200x). (C, D): Stromal negativity for CD4 positivity for CD4 (200x). (E, F) Variable expression of TNF-α, with low and high expression (200x). (G, H) Low and high expression of COX-2 (200x) in OSCC samples.

The expression of inflammatory markers varied, with a low expression of CD4 (mean: 5.32%, minimum: 1%, and maximum: 32.4%) and CD68 (mean: 4.65%, minimum: 1%, and maximum: 15.8%). The mean of TNF-α expression was 12.4% (minimum: 1%, maximum: 45.3%). The expression of COX-2 was higher, with a maximum of 65.7% (Table 2 and Figure 2). In addition, regarding the labelling pattern, CD68 demonstrated cytoplasmic staining in the macrophages, with higher intensity in the peritumoral regions and variable expression (Figures 2A and 2 B). CD4 showed both membranous and cytoplasmic staining in the lymphocytes, predominantly located within the peritumoral inflammatory infiltrates (Figures 2C and 2D). TNF-α exhibited cytoplasmic and perinuclear staining in both tumor and immune cells, with more prominent expression in tumor-infiltrating immune cells (Figures 2E and 2F). COX-2 displayed cytoplasmic expression, with stronger intensity observed at the tumor–stroma interface (Figures 2G and 2H).

**Table 2:** Analysis of the association between the expression of inflammatory markers and prognostic variables (tumor size, nodal metastasis, and clinical stage).

Marker (%)	n (%)	T (size)	Nodal metastasis	Clinical stage
		p-value	p-value	p-value
CD68				
≤ 4.5	40 (58.8)			
≥ 4.6	28 (41.2)	0.48	0.20	0.60
CD4				
≤ 5	45 (65.2)	0.37	0.67	0.81
≥ 6	24 (34.8)			
Cyclooxygenase (COX)-2				
≤ 12	47 (69.1)	0.01*		
≥ 13	21 (30.9)		0.50	0.80
Tumor necrosis factor (TNF)-α				
≤ 12	36 (58.1)	0.54	0.44	0.16
≥ 13	26 (41.9)			

^*^Significant p-value; Cramer’s V = 0.30

Table 2 shows the association analysis between the prognostic variables (tumor size, nodal metastasis, and clinical stage) and these markers. Only COX-2 was associated with tumor size (T) (p = 0.01). The expression of CD4 and CD68 was not associated with any other clinical variables or tumor differentiation. However, high expression of TNF-α was observed in moderately to poorly differentiated tumors (p = 0.025). No association was observed with CD68, CD4, TNF-α, or COX-2 and multifocal neural involvement, which was noted in 39% of the cases (n = 28).

**Figure 3:**
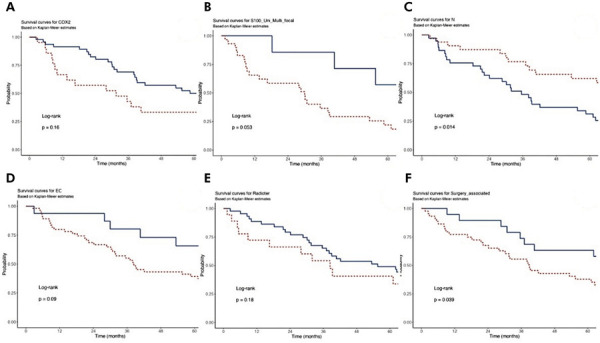
Univariate Log-rank analysis in 5-year DDS. (A) High and low expression of COX-2. (B) Perineural invasion (uni or multifocal). (C) Nodal involvement. (D) Clinical stage. (E) Treatment with radiotherapy. (F) Surgery associated with chemo or radiotherapy. A significant reduction was noted in 5-year DSS in patients with nodal involvement (p = 0.01). A significant increase in 5-year DSS was noted in patients who had undergone surgery in association with chemo/radiotherapy (p = 0.03).

Table 3 and Figure 3 show the results of the DSS analysis. The following factors were associated with a higher or lower risk of death in the univariate regression analysis: regional lymph nodes (hazard ratio [HR]: 0.47, confidence interval [CI]: 0.25–0.87, p = 0.001; lower risk in the absence of nodal involvement); surgery without radiotherapy or chemotherapy (HR: 2.09, CI: 1.02–4.27, p = 0.043; with higher risk in the absence of adjuvant treatment. COX-2 (HR: 1.55, CI: 0.84–2.85, p = 0.163), neural invasion (HR: 2.76, CI: 0.95–8.03, p = 0.063), and clinical stage (HR: 1.92, CI: 0.89–4.13, p = 0.09, stages III and IV), radiotherapy (HR: 1.57, CI: 0.81–3.04, p = 0.179) were included in the multivariate analysis, considering the 0.2 as the cut-off.

**Table 3:** Hazard ratio associated with disease-specific survival in squamous cell carcinoma cases with clinical and inflammatory markers.

Variables	Univariate analysis HR (95%CI)	p-value	Multivariate analysis HR (95%CI)	p-value
Age (years)				
≤ 50	Reference			
≥ 51	1.05 (0.51–2.18)	0.890	-	-
Sex				
Male	Reference		-	-
Female	0.87 (0.44–1.71)	0.688		
Tumor (T)				
1–2	Reference			
3–4	1.27 (0.69–2.32)	0.442	-	-
Regional lymph nodes				
No	0.47 (0.25–0.87)	0.001	0.61 (0.18–2.04)	0.424
Yes	Reference			
Clinical stage				
I + II	Reference			
III + IV	1.92 (0.89–4.13)	0.09	1.93 (0.40–9.33)	0.415
Radiotherapy (RT)				
Yes	Reference			
No	1.57 (0.81–3.04)	0.179	0.85 (0.24–3.02)	0.799
Chemotherapy (CT)				
Yes				
No	0.93 (0.50–1.76)	0.834	-	-
Surgery +CT or RT				
Yes				
No	2.09 (1.02–4.27)	0.043	3.12 (0.82–11.93	0.095
Multifocal perineural invasion (PNI)				
Yes	2.76 (0.95–8.03)	0.063	3.55 (0.93–13.50)	0.063
No				
CD68 (%)				
≤ 4.5				
≥ 4.6	0.89 (0.49–1.61)	0.699	-	-
CD4 (%)				
≤ 5				
≥ 6	0.71 (0.38–1.33)	0.282	-	-
Cyclooxygenase (COX)-2 (%)				
≤ 12				
≥ 13	1.55 (0.84–2.85)	0.163	5.15 (1.55–17.12)	0.008*
TGFα (%)				
≤ 12				
≥ 13	097 (0.52-1.81)	0.932	-	-

*Statistically significant. Bold variables were included in the multivariate analysis (p ≤ 0.20).

Multivariate Cox regression model identified high expression of only COX-2 (HR: 5.15, CI: 1.55–17.12, p = 0.008) as an independent prognostic factor. Notably, multifocal neural invasion demonstrated only borderline significance in both univariate and multivariate survival analyses (p = 0.063).

## Discussion

A functional association between malignancy and chronic inflammation has been described previously. The establishment of a TME can favor reciprocal interactions between developing tumor cells and stromal cells, promoting tumor progression.^14^ Inflammatory mediators can accumulate in the TME, induce cell proliferation, and promote prolonged cell survival by activating oncogenes and inhibiting tumor suppressor genes.^15^ Tumor-associated inflammatory cells include macrophages, lymphocytes, neutrophils, NK/NKT (natural killer T) cells, mast cells, neutrophils, and lymphocytes (T and B), as well as mediators such as cytokines, prostaglandins, chemokines, reactive oxygen species, and nitrogen radicals.^14,15^

CD68+ macrophages, particularly M2-polarized TAMs, contribute to immunosuppression and extracellular matrix remodeling. Their activity is shaped by TNF-α and COX-2 signaling, forming a tumor-promoting cycle.^3^ Similarly, CD4+ T cells can exert anti-tumor (Th1) or pro-tumor (Th2/Treg) effects depending on the cytokine milieu, with TNF-α acting as a key modulator that can promote inflammation or immune evasion.^4,5^ TNF-α serves as a central link between chronic inflammation and tumor progression, driving macrophage polarization and upregulating COX-2, consequently sustaining inflammation, supporting immune suppression, and facilitating angiogenesis.^6^ COX-2 further amplifies this loop by enhancing the recruitment and activation of CD68+ TAMs, thereby reinforcing the pro-TME.^7,8^

TAMs are abundant in the TME and are considered important for cancer progression.^16^ TAMs may exhibit two phenotypes, M1 or M2, depending on the cytokines, chemokines, and other receptors/mediators.^3^ M1 and M2 TAMs exhibit anti-tumor and pro-tumor properties, respectively. M2 macrophages express CD68 and promote immunosuppression, angiogenesis, tumor invasion, and metastasis.^17^ Certain studies have reported an association between the expression of CD68+ TAM and the prognosis of OSCC.^18-20^ However, similar to our results, a meta-analysis of four studies revealed no association between high numbers of CD68+ TAMs and the overall survival of patients with OSCC.^21^ The lack of prognostic significance for CD68 in our cohort may reflect the heterogeneity of TAM polarization in OSCC or the influence of other TME components, such as TNF-α or COX-2, that may override individual macrophage effects.

CD4+ cells have been proposed as prognostic biomarkers in patients with OSCC^22,23^. The function of CD4+ lymphocytes in the TME is not completely understood, especially considering the range of CD4+ cell subsets with distinct functions, from Th1 cells stimulating cytotoxic cell responses to regulatory T cells suppressing the immune system.^22^ A study evaluating CD4 expression in primary OSCC observed a significant negative correlation with the TNM stage.^23^ Another study demonstrated that CD4+ expression was related to overall OS and progression-free survival in univariate analysis. Low CD4 expression was found to identify early-stage OSCC with poor prognosis^24^. However, our results did not corroborate these associations, possibly because of the predominance of advanced-stage tumors in our cohort, in which CD4+ subsets may have been functionally exhausted or overshadowed by immunosuppressive networks. Different analyses related to CD4 exist, and controversial results regarding survival, CD4, and OSCC.^4,5^

Elevated expression of TNF-α in the TME has been associated with tumoral invasion through enhanced pro-inflammatory processes in OSCC cells and paracrine-mediated recruitment and activation of inflammatory cells.^6^ Its function as a pro-inflammatory cytokine can either facilitate cancer progression or function as a potential cancer inhibitor, considering its pro-apoptotic effects.^25^ These scenarios make it difficult to assign a definitive prognosis to TNF-α. Although it is associated with OSCC and head and neck cancer, only a few studies related to TNF-α and survival or with OSCC and prognostic variables are available.^2^ An analysis of TNF-α by immunohistochemistry demonstrated that this marker influenced the survival of patients with OSCC.^26^ contrary to our results. Although this marker was not an independent prognostic factor in our sample, we observed its high expression in moderately/poorly differentiated tumors. Although TNF-α was not an independent prognostic factor in our cohort, its high expression in moderately/poorly differentiated tumors suggests a context-dependent role, potentially promoting aggression in certain histological subtypes but not universally impacting survival.

COX-2, a prostaglandin-endoperoxide synthase 2 enzyme, generates prostanoids such as prostaglandin E2, which modulate carcinogenic effects.^27^ It is commonly expressed in different types of cancer and plays multifaceted roles in carcinogenesis and treatment resistance. COX-2 is released into TME by fibroblasts (CAFs), macrophages (M2), and cancer cells. It promotes cancer stem cell-like activity and contributes to inflammation, proliferation, apoptosis, angiogenesis, invasion, and metastasis. In addition, COX-2-mediated hypoxia in the TME, along with its positive interactions with YAP1 and anti-apoptotic factors, collectively contributes to cancer cell resistance to chemotherapeutic drugs.^28^

COX-2 emerged as a critical prognostic marker in our study, with high expression significantly associated with a larger tumor size (p = 0.01). This finding aligns with the proposed role of COX-2 in promoting tumor growth through prostaglandin-mediated proliferation, angiogenesis, and suppression of apoptosis.^27,28^ The correlation between COX-2 and advanced T-stage suggests that COX-2 may drive the local expansion of OSCC, potentially through interactions with CAFs and immune cells in the TME.^7^ This reinforces the rationale for targeting COX-2 in adjuvant therapies, particularly in patients with large tumors, as its inhibition could disrupt tumor-promoting signaling pathways.^28-31^

Our finding that COX-2 is an independent prognostic factor for poor DSS underscores its potential as a therapeutic target. Similarly, patients with N2-stage OSCC and high local COX-2 expression have a significantly worse prognosis/survival.^29^ A systematic review demonstrated controversial results in different studies, with some supporting COX-2 as a predictor of OSCC prognosis, whereas others presented opposite results.^8^ These conflicting results may be explained by differences in the detection of COX-2, types of survival outcomes, sample sizes, patient selection, scoring systems, and different antibodies used.^30^ However, our data, combined with evidence from other cancers, suggest that COX-2 inhibition warrants further exploration in OSCC, particularly in advanced-stage disease.

The application of COX-2 inhibitors in lung, colon, breast, and prostate cancers appears to reduce cancer risk.^31^ However, several points must be highlighted: certain types of cancers are resistant to COX-2 inhibitors, even the expression is controversial depending on the site and tumor type; both the suppression and activation of COX-2 are associated with tumorigenesis, among other issues. The unregulated activation of COX-2 is associated with a worse prognosis for most types of cancer. Considering this, the suppression of COX-2 could be considered a promising approach, particularly as an adjuvant therapy.^28^

An interesting aspect of our study was the inclusion of the PNI as a prognostic variable. Our univariate analysis revealed multifocal PNI as a prognostic variable, although it was not a prognostic factor in multivariate analysis. PNI is recognized as an unfavorable prognostic factor in several solid malignancies, as well as in OSCC^32^. The lack of significance in the multivariate analysis may reflect the dominant impact of COX-2 and tumor stage in our cohort; however, PNI remains a clinically relevant feature that warrants further investigation.

Our analysis revealed that patients who underwent surgery with adjuvant radiotherapy or chemotherapy (combination therapy) had a higher risk of mortality. Thus, it is essential to consider the inherent selection bias in retrospective studies, as patients receiving adjuvant therapy often present with advanced-stage disease, perineural invasion, or other high-risk features that necessitate multimodal treatment, but also correlate with poorer prognosis. Thus, the observed association possibly reflects disease aggressiveness rather than the detrimental effects of the therapy itself. This may imply that although adjuvant regimens improve locoregional control in high-risk OSCC, they fail to fully overcome the survival disadvantages associated with advanced disease. Future prospective studies stratifying patients according to risk factors are required to elucidate the independent effects of treatment modalities on survival.

This study has certain limitations, including a small sample size, use of a single Brazilian cohort, retrospective design, and lack of control for treatment variations, which may affect the generalizability and strength of the findings. In addition, the absence of functional assays limits causal interpretation, and validation in independent cohorts is required. These findings are especially relevant considering the limitations of the World Health Organization histological grading system, which is subjective and fails to capture the biological complexity of tumors. Integrating immune and inflammatory markers into prognostic models may improve accuracy and offer a more comprehensive understanding of disease progression. This overrepresentation of advanced-stage OSCC may have influenced the observed associations and underscores the need for future research with a more balanced clinical stage distribution to validate the prognostic value of inflammatory markers.

In summary, our data demonstrated that the expression of the markers CD68, CD4, and TNF-α was not associated with DSS in OSCC in our Brazilian sample. In contrast, high COX-2 expression was associated with tumor size and was identified as an independent prognostic factor correlating with decreased survival. In addition, elevated expression of TNF-α was observed more in moderately to poorly OSCC.

## Data Availability

The authors declare that all data generated or analyzed during this study are included in this published article.

## References

[1] Sung H, Ferlay J, Siegel RL, Laversanne M, Soerjomataram I, Jemal A (2021). Global Cancer Statistics 2020: GLOBOCAN estimates of incidence and mortality worldwide for 36 cancers in 185 countries. CA Cancer J Clin.

[2] Niklander SE (2021). Inflammatory mediators in oral cancer: pathogenic mechanisms and diagnostic potential. Front Oral Health.

[3] Alves AM, Diel LF, Lamers ML (2018). Macrophages and prognosis of oral squamous cell carcinoma: a systematic review. J Oral Pathol Med.

[4] Nguyen N, Bellile E, Thomas D, McHugh J, Rozek L, Virani S, Head and Neck SPORE Program Investigators (2016). Tumor infiltrating lymphocytes and survival in patients with head and neck squamous cell carcinoma. Head Neck.

[5] Balermpas P, Michel Y, Wagenblast J, Seitz O, Weiss C, Rödel F (2014). Tumour-infiltrating lymphocytes predict response to definitive chemoradiotherapy in head and neck cancer. Br J Cancer.

[6] Goertzen C, Mahdi H, Laliberte C, Meirson T, Eymael D, Gil-Henn H (2018). Oral inflammation promotes oral squamous cell carcinoma invasion. Oncotarget.

[7] Frejborg E, Salo T, Salem A (2020). Role of cyclooxygenase-2 in head and neck tumorigenesis. Int J Mol Sci.

[8] Biasin FF, Franco AM, Louzeiro GC, Cherubini K, Salum FG (2024). Association of COX-2, TNF-a, TLR4, and IKKa with survival of patients with oral squamous cell carcinomas: a systematic review. Asian Pac J Cancer Prev.

[9] Beegum F, Trimukhe A (2024). Traversing the terrain: potential pitfalls within the AJCC 8th edition staging system for lip and oral cavity cancers. Head Neck Pathol.

[10] Muller S, Tilakaratne WM (2022). Update from the 5th Edition of the World Health Organization Classification of head and neck tumors: tumours of the oral cavity and mobile tongue. Head Neck Pathol.

[11] Pai R, Karki S, Agarwal R, Sieber S, Barasch S (2022). Optimal settings and clinical validation for automated Ki67 calculation in neuroendocrine tumors with open source informatics (QuPath). J Pathol Inform.

[12] Kakushadze Z, Yu W (2017). *K-means and cluster models for cancer signatures. Biomol Detect Quantif.

[13] Aivazian K, Ebrahimi A, Low TH, Gao K, Clifford A, Shannon K (2015). Perineural invasion in oral squamous cell carcinoma: quantitative subcategorisation of perineural invasion and prognostication. J Surg Oncol.

[14] Hanahan D, Weinberg RA (2011). Hallmarks of cancer: the next generation. Cell.

[15] Feller L, Altini M, Lemmer J (2013). Inflammation in the context of oral cancer. Oral Oncol.

[16] Galdiero MR, Garlanda C, Jaillon S, Marone G, Mantovani A (2013). Tumor associated macrophages and neutrophils in tumor progression. J Cell Physiol.

[17] Mantovani A, Biswas SK, Galdiero MR, Sica A, Locati M (2013). Macrophage plasticity and polarization in tissue repair and remodelling. J Pathol.

[18] Ni YH, Ding L, Huang XF, Dong YC, Hu QG, Hou YY (2015). Microlocalization of CD68+ tumor-associated macrophages in tumor stroma correlated with poor clinical outcomes in oral squamous cell carcinoma patients. Tumour Biol.

[19] Takahashi H, Sakakura K, Kudo T, Toyoda M, Kaira K, Oyama T (2017). Cancer-associated fibroblasts promote an immunosuppressive microenvironment through the induction and accumulation of protumoral macrophages. Oncotarget.

[20] Kikuchi M, Yamashita D, Hara S, Takebayashi S, Hamaguchi K, Mizuno K (2021). Clinical significance of tumor-associated immune cells in patients with oral squamous cell carcinoma. Head Neck.

[21] Chohan MH, Perry M, Laurance-Young P, Salih VM, Foey AD (2023). Prognostic role of CD68 + and CD163 + tumour-associated macrophages and PD-L1 expression in oral squamous cell carcinoma: a meta-analysis. Br J Biomed Sci.

[22] Ruiter EJ, Ooft ML, Devriese LA, Willems SM (2017). The prognostic role of tumor infiltrating T-lymphocytes in squamous cell carcinoma of the head and neck: a systematic review and meta-analysis. OncoImmunology.

[23] Jeyapriya SM, Mohan AM, Kumar MS, Nirmal RM (2024). Expression of CD4+ and CD8+ tumor-infiltrating lymphocytes in oral squamous cell carcinoma and their relationship with clinicopathological parameters: a cross-sectional study. Cureus.

[24] Wongpattaraworakul W, Choi A, Buchakjian MR, Lanzel EA, Kd AR, Simons AL (2024). Prognostic role of tumor-infiltrating lymphocytes in oral squamous cell carcinoma. BMC Cancer.

[25] Wang X, Lin Y (2008). Tumor necrosis factor and cancer, buddies or foes?. Acta Pharmacol Sin.

[26] Dantas TS, Barros Silva PG, Lima Verde ME, Ribeiro AL, Cunha MD, Mota MR (2019). Role of inflammatory markers in prognosis of oral squamous cell carcinoma. Asian Pac J Cancer Prev.

[27] Li F, Zhu YT (2015). HGF-activated colonic fibroblasts mediates carcinogenesis of colonic epithelial cancer cells via PKC-cMET-ERK1/2-COX-2 signaling. Cell Signal.

[28] Hashemi Goradel N, Najafi M, Salehi E, Farhood B, Mortezaee K (2019). Cyclooxygenase-2 in cancer: a review. J Cell Physiol.

[29] Sano Y, Kogashiwa Y, Araki R, Enoki Y, Ikeda T, Yoda T (2018). Correlation of inflammatory markers, survival, and COX2 expression in oral cancer and implications for prognosis. Otolaryngol Head Neck Surg.

[30] Sakurai K, Urade M, Noguchi K, Hashitani S, Takaoka K, Segawa E (2007). Prognostic significance of cyclooxygenase-2 and DNA topoisomerase IIalpha expression in oral carcinoma. Head Neck.

[31] Brizzolara A, Benelli R, Venè R, Barboro P, Poggi A, Tosetti F The ErbB family and androgen receptor signaling are targets of Celecoxib in prostate cancer.

[32] Spoerl S, Spoerl S, Reil S, Gerken M, Ludwig N, Taxis J (2022). Prognostic value of perineural invasion on survival and recurrence in oral squamous cell carcinoma. Diagnostics (Basel).

